# RETRACTED ARTICLE: Analysis of coastal rainfall pattern based on artificial intelligence and global cultural communication

**DOI:** 10.1007/s12517-021-08004-3

**Published:** 2021-08-13

**Authors:** Liang Huaping

**Affiliations:** 1 Xi’an Jiaotong University, Xi’an, 710049 Shaanxi China; 2 Xi’an Technological University, Xi’an, 710021 Shaanxi China

**Keywords:** Artificial intelligence, Coastal rainfall, Rain pattern analysis, Globalization, Cultural communication

## Abstract

Artificial intelligence has been applied in all aspects of cultural industry, and has affected the micro, meso, and macro level of cultural industry, accelerated the transformation of cultural industry, and changed the structural system of cultural development. Based on artificial intelligence technology, this paper studies 52 years of coastal rainfall and analyzes the characteristics of rainfall pattern by observing the latest meteorological observation data in southern China from 1969 to 2020. Based on the analysis of rain patterns in South China, it is found that only 8% of the annual precipitation in a southern region of China has a significant increasing trend. The monthly trend showed the most obvious downward and upward trend in April and November. Finally, by analyzing the results of artificial intelligence research, we can create a model of global cultural communication system based on artificial intelligence application. The logic model combines the elements of power, system, service and research framework knowledge, and puts forward the idea of system optimization based on artificial intelligence application to solve subsystem problems; For the service subsystem, artificial intelligence should be used to strengthen the supply and demand of services; For knowledge subsystem, artificial intelligence is needed to improve education and training skills, including knowledge service skills and knowledge management skills. The global cultural communication system must use artificial intelligence to improve the synergy of subsystems. Through the analysis of coastal rainfall pattern and the study of global cultural communication based on artificial intelligence, this paper applies it to cultural communication and promotes the development of global cultural communication.

## Introduction

As an important part of weather and hydrological cycle, rainfall has changed in recent years due to the impact of global climate change. The spatial and temporal distribution of rainfall restricts the social and economic development of the region to a certain extent, and plays an important role in preventing regional floods, resisting drought, and effectively using water resources (Rafique et al. 2015). Based on the decomposition of artificial intelligence orthogonal function method, this paper analyzes the Precipitation Law and dominant rainfall pattern of coastal rainfall. The results show that the two precipitation models, which are dominated by coastal rainfall, have strong annual signals. The spatial interpretation rate of the first model is 60.09%, the basic spatial distribution is consistent with the annual average precipitation distribution, and shows synchronous changes in the whole process. From the regional analysis, according to the monthly average time coefficient, the positive and negative peak values in June and December show that the spatial interpretation rate of the second model is 11.41%, and the spatial distribution gradually decreases from south to north. The monthly average time coefficient has positive and negative peaks in September and March. The third and fourth models have strong signals for half a year, and their spatial interpretation rates are less than 5%. According to the results of precipitation pattern analysis based on artificial intelligence technology, this paper analyzes the reasons of the multifaceted background field of precipitation spatial-temporal distribution in southern China from the perspective of water vapor flux in the reanalysis of historical data (Vikas et al. 2013). The results show that the propagation of water vapor in Northeast China mainly occurs in late autumn and winter (Rashid et al. 2018). Generally speaking, it does not bring more rainfall in southern China. The flood season in southern China comes from the summer rainfall accompanied by the Indian Ocean, and its water vapor flow and southwest water vapor flow back to the South China Sea (Martinez-Mier 2018). Finally, based on the application of artificial intelligence, this paper makes a comprehensive study on the improvement of global cultural communication, using artificial intelligence to enhance the ability of subsystems, empower knowledge subsystems, change political and legal subsystems, integrate subsystem services, and strengthen the relationship between subsystems. The synergy of the system makes it possible to improve and benefit each other and create new functions beyond the functions of each subsystem (Mohan et al. 2017). The popularity of COVID-19 in early 2020 will have a significant impact on offline cultural consumption, thus making the growth momentum of cultural transmission seriously inadequate. In this case, we must use the application of artificial intelligence to promote the intelligent, digital, and network development of cultural industry, so as to reverse the offline crisis of online opportunities (Thivya et al. 2017). Specifically, the power subsystem needs to change the technology application, market competition, marketing and consumption in cyberspace, and improve the intelligence level of these four power elements; The political and legal subsystem must have a deep understanding of culture, consumers, and related affairs (Nagarajan et al. 2010). Research institutions, relevant service organizations and knowledge of AI needs, formulate practical plans for AI applications, and create a political and legal environment conducive to AI applications (Purushotham et al. 2011); The service platform subsystem needs to support the application of artificial intelligence in the cultural industry in the aspects of project penetration, investment and financing, innovation, and entrepreneurship; Knowledge subsystem should use artificial intelligence to strengthen knowledge heritage, knowledge innovation and space value creation, improve online cultural creation, dissemination and consumption information, and deeply integrate the innovation chain, industry chain, and network value chain of cultural industry, so as to drive the development of global cultural communication (Muhammad et al. 2018).

## Materials and methods

### Data source

The coverage of the study area is 108 ° east longitude to 118 ° and latitude 18 ° north to 26 °, including all areas of H and G provinces. The region is located in the difficult part of China and is connected with the warm South China Sea. It is heavily affected by tropical cyclones, Asian summer monsoon and tropical trough. The region is the wettest in China and also the most vulnerable to hurricanes. The study of Precipitation Evolution in this area is of great significance for the economic and social development of this area (Samanta et al. 2013).

Using the reanalysis data provided by NOAA and NCEP, the spatial pattern of precipitation distribution in southern China is analyzed and understood. The data storage format is NetCDF, which is widely used to store atmospheric data, such as meteorology and hydrology. It has good stability, and is easy to manage and access. In this paper, the monthly average reanalysis data of zonal wind, meridional wind (U, V wind), and specific elements of meteorological humidity are used. The data covers the period from January 1969 to December 2020, with a spatial resolution of 2.5 ° × 2.5 °, East longitude 0 ° to 357.5 °, 90 n ° to 90 s °, Use 144 × 73 grid division of the world. The monthly mean data set of meteorological elements includes four dimensions: longitude, latitude, isobaric surface (from 1000 HPA near the ground to 300 HPA above sea level), and monthly dimension.

### Trend analysis of coastal rainfall

Mann Kendall trend test method is widely used to determine whether meteorological data and hydrological data change significantly with time.

1 $$ T={\sum}_{i=1}^{n-1}\kern0.1em {\sum}_{j=i+1}^n\kern0.1em \operatorname{sgn}\left({x}_j-{x}_i\right) $$

sgn(xj-xi):

2 $$ \operatorname{sgn}\left({x}_j-{x}_i\right)=\left\{\begin{array}{c}+1\ {x}_j-{x}_i>0\\ {}0{x}_j-{x}_i=0\\ {}-1{x}_j-{x}_i<0\end{array}\right. $$

In the randomized controlled study, if the sample data does not conform to the trend, then t follows the normal distribution, and the corresponding mean value is 0. The difference is calculated as follows:

3 $$ \mathrm{Var}(T)=\frac{n\left(n-1\right)\left(2n+5\right)-{\sum}_{k=1}^p\kern0.1em {q}_k\left({q}_k-1\right)\left(2{q}_k+5\right)}{18} $$

To calculate the statistical value of ZMK test according to the normal distribution, refer to the following contents:

4 $$ {Z}_{mk}=\left\{\begin{array}{cc}\frac{T-1}{\sqrt{\mathrm{Var}(T)}}& T>0,\\ {}0& T=0,\\ {}\frac{T+1}{\sqrt{\mathrm{Var}(T)}}& T<0.\end{array}\right. $$

The value of P can be understood as the probability of a low probability event, and its value is calculated as follows:

5 $$ P=2\left(1-\frac{1}{\sqrt{2\pi }}{\int}_{-\infty}^{\left|{z}_{mk}\right|}\kern0.1em {e}^{-\frac{x^2}{2}} dx\right) $$

### Evaluation of different rainfall patterns

Nowadays, the precipitation models commonly used in design at home and abroad includes Chicago precipitation model and P.C. precipitation model. Because the Chicago precipitation model is based on the rainstorm formula, it is easy to determine the precipitation intensity at different times under different return time periods and maximum aspect ratio, so as to obtain the precipitation process designed for different periods, and obtain the corresponding precipitation model effect, which is more reflective (Sezgin et al. 2018). Typical characteristics of most precipitation PC rainfall design is based on long series and short data about precipitation history. Historical data should be analyzed and processed to obtain precipitation distribution ratio and calculate precipitation classification design process (Shahid et al. 2019). Even if the obtained rainfall term is closer to the actual rainfall process, the process is difficult and not easy to change the current peak time, and does not meet the rainfall requirements of this scheme. Bi and other researchers pointed out that the distribution of precipitation in X city is uneven and concentrated, and the precipitation is basically unbalanced (Singh et al. 2017). Under the comprehensive consideration, this paper chooses the Chicago precipitation model as the precipitation design. According to the existing data, the formula of rainstorm intensity in X city is as follows:

6 $$ q=\frac{167A\times \left(1+C\times \lg P\right)}{{\left(t+b\right)}^n} $$

The final general formula (7) of rainstorm intensity in XX new area.

7 $$ q=\frac{2210.87\times \left(1+2.915\times \lg P\right)}{{\left(t+21.933\right)}^{0.974}} $$

## Results

### Trend analysis of coastal rainfall

**Fig. 1.**
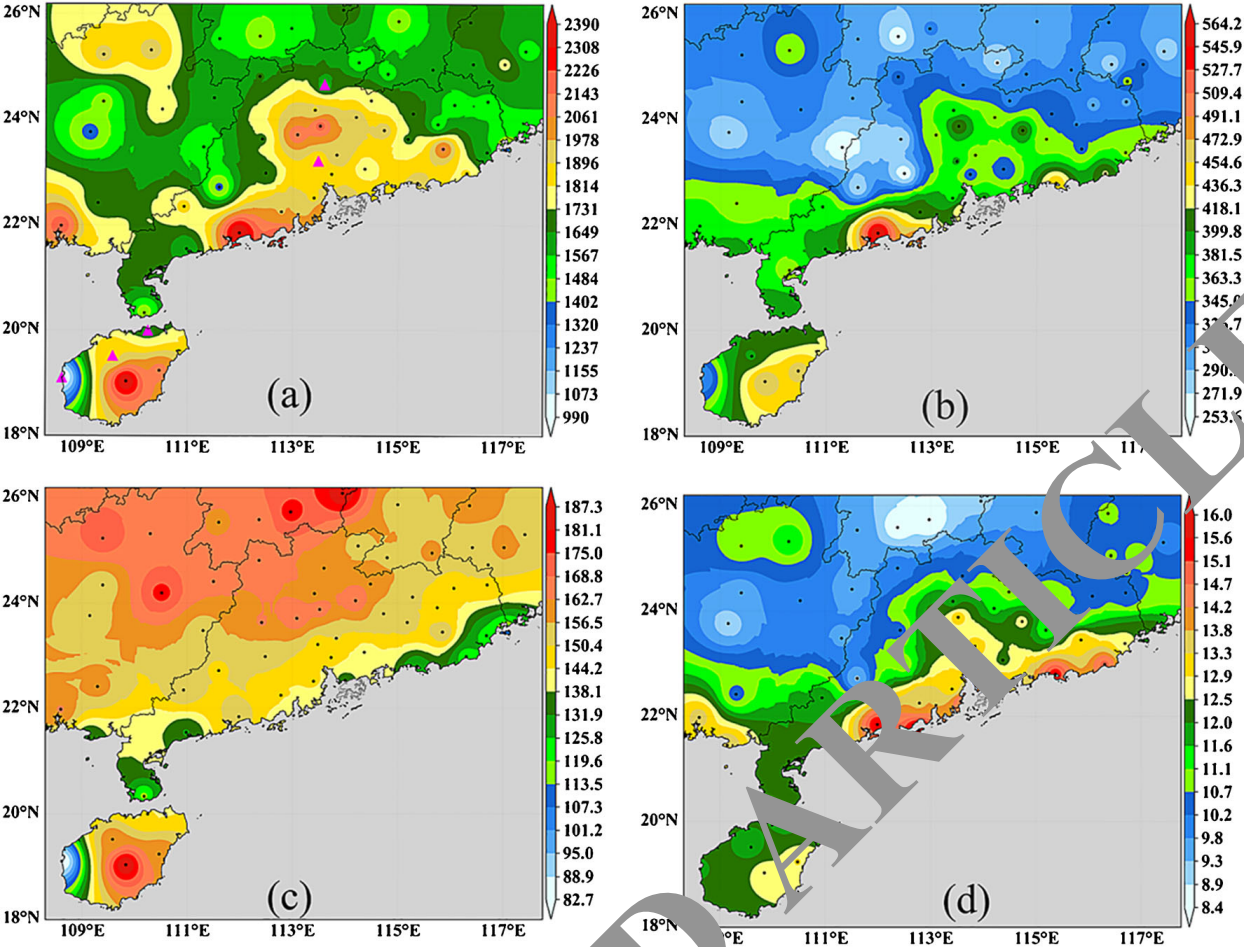
Spatial distribution of annual rainfall characteristics in South China from 1969 to 2020

Figure 1a shows the spatial distribution of average annual precipitation in southern China from 1969 to 2020, and Fig. 1b shows the spatial distribution of standard deviation of annual precipitation in southern China. Figures 1a and b show that the areas with the highest rainfall are located in the central and southern coastal areas of G Province, which is also the area with the largest deviation from the standard year. Secondly, the coastal areas of southeast province h and Southeast Region G are also areas with high rainfall.

Table 1 lists the detailed information of some representative stations in the annual indicators, including annual average precipitation, standard deviation of annual precipitation, days of annual precipitation and annual average precipitation intensity. From 1969 to 2020, the meteorological stations with the highest and lowest rainfall in three years are selected. S and G are the two most populous cities in the study area. Table 1 shows the details of the national standard weather stations in these two cities.

**Table 1:** Annual rainfall information of some representative meteorological stations

Station name	Provincial capital	Longitude	Latitude	Ave	Std	Rds	Pi
Long	City F	117	25.1	1756.58	331.1	160	11
Guang	City G	113.5	23.2	1 809.58	384.3	146.9	12.3
Shen	City G	114	22.5	1920.65	384	135.1	14.2
Yang	City G	112	21.9	2390.2	564.2	15456	15.5
Wu	City G	11130	23.5	1483.48	253.7	153.1	9.69
Qin	City G	108.6	22	2193.99	381.6	163.1	13.5
Dong	City H	108.6	19.1	990.28	307.4	82.73	12
Qiong	City H	109.8	19	2384.53	472.7	187.3	12.7
Chen	City H	113	25.7	1496.82	310.1	177.2	8.45

**Fig. 2.**
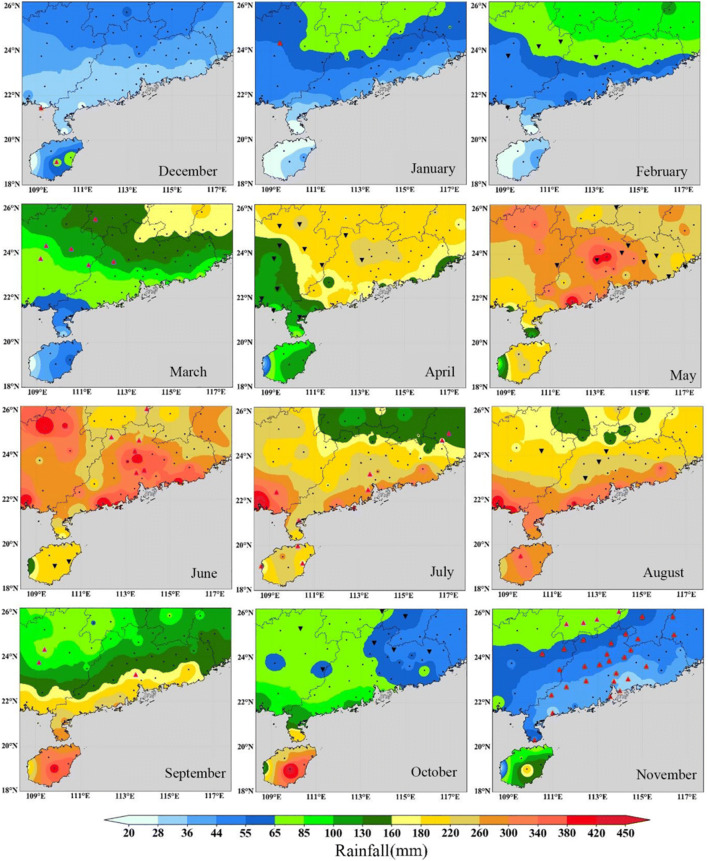
Spatial distribution of monthly average rainfall in South China from 1969 to 2020

According to the daily precipitation data of 63 major national base stations in South China, the monthly precipitation of each station in the region is calculated as a cumulative amount, and the average monthly precipitation of each season in 52 years is calculated, and the average is weighted by inverse distance. The spatial distribution model of average monthly precipitation in southern China is obtained by using spatial coupling method. From 1969 to 2020. Among them, November to February of the next year is a dry month in southern China, with an average monthly rainfall of 20–85mm. April to August is the rainy season in southern China, with an average monthly rainfall of 130–450mm. The wettest month is June, with an average monthly rainfall of 220–450 mm. In most areas, the rainfall distribution is 400 m. In addition, the average rainfall in other months is about 55–260mm. It is shown in Fig. 2.

**Fig. 3.**
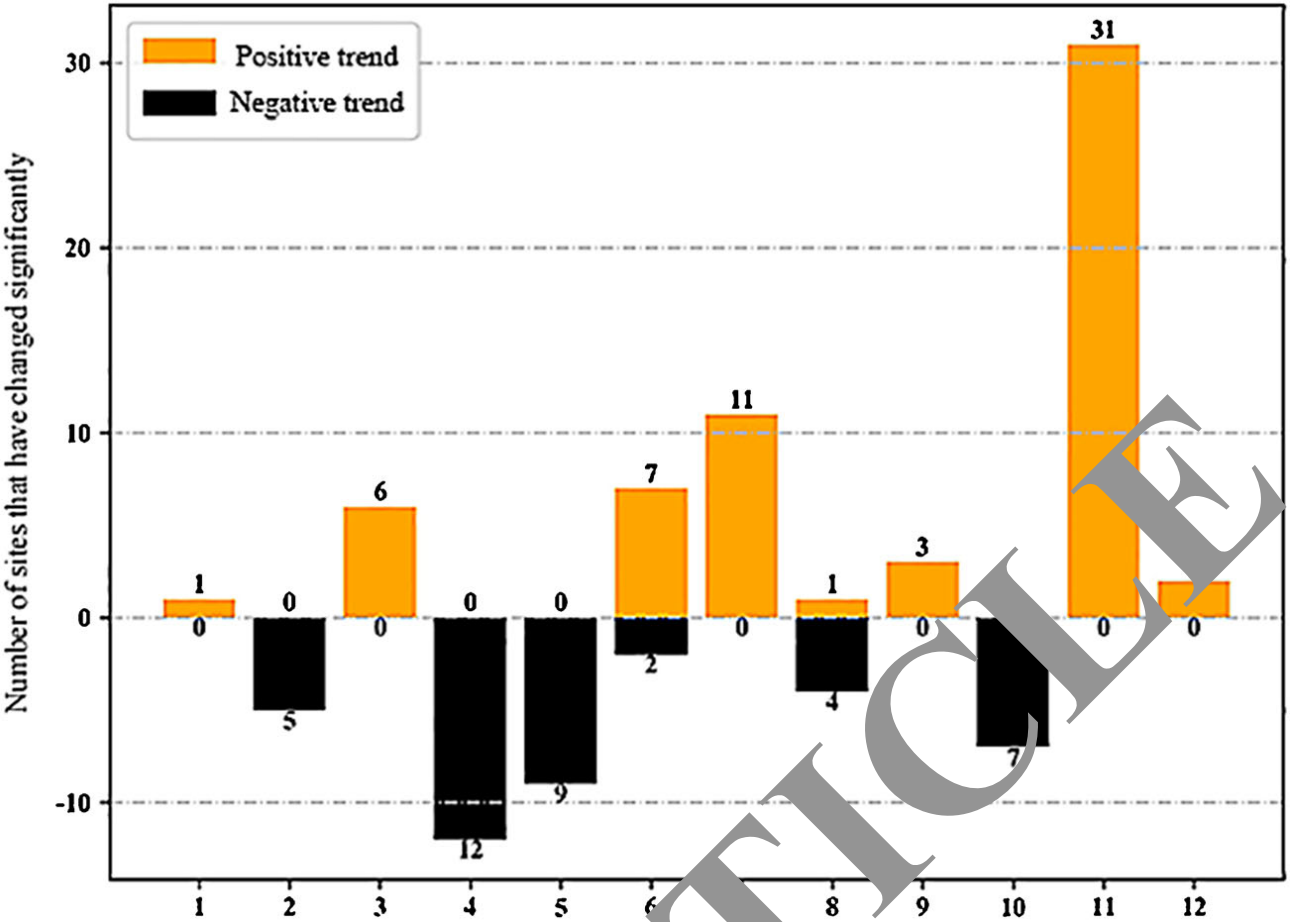
Number of stations with significant monthly rainfall variation (confidence level above 90%) in different months in recent years

It can be seen from Fig. 3 that November is the month with the largest number of stations with significant positive trend of monthly precipitation, while April is the month with the largest number of stations with significant negative trend of monthly precipitation.

Based on the monthly precipitation series of 63 basic weather stations in southern China from 1969 to 2020, the monthly precipitation anomaly matrix is created to analyze the orthogonal empirical function, and the contribution rate of each spatial model and the contribution of square difference are obtained (see Table 2). It can be seen from Table 2 that the cumulative contribution rate of the first four spatial models (eof1–eof4) has changed by nearly 80%, and the eigenvalues of the corresponding models have also passed the North test. EOF decomposition is the main spatial model of precipitation in southern China, and each model is independent and variable.

**Table 2:** Variance contribution rate and cumulative variance contribution rate of each spatial mode (EOF1–EOF6)

EOFs	EOF1	EOF2	EOF3	EOF4	EOF5	EOF6
Variance contribution rate (%)	60.1	11.4	4.22	3.88	2.31	1.74
Cumulative variance contribution rate (%)	60.1	71 50	75.7	79.6	81.9	83.7

**Fig. 4.**
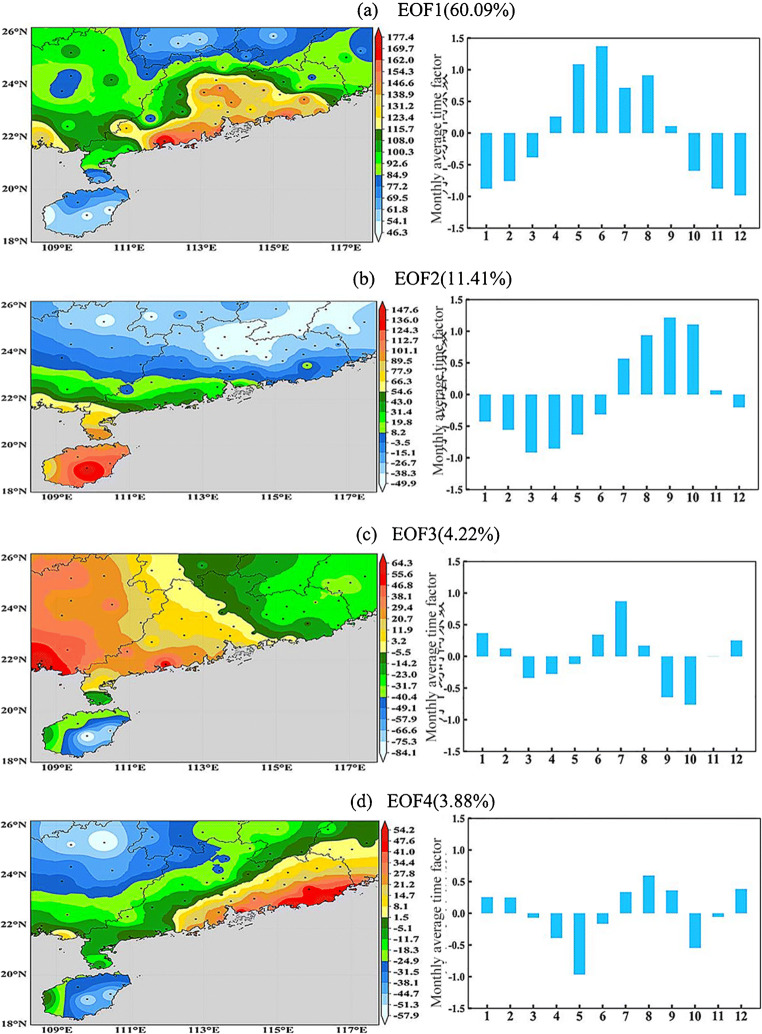
Spatial distribution and monthly average time coefficient of EOF1–EOF4 rainfall modes in South China

Figure 4 shows the first mock exam (EOF1) to fourth pattern (EOF4) spatial distribution of monthly precipitation. Comparing the monthly precipitation spatial pattern in Fig. 4, we can see that the observed monthly precipitation pattern is different from the decomposed EOF spatial model eof1–eof4. This is an approximate spatial pattern, but there are many different spatial patterns. This is because the spatial pattern of monthly precipitation is usually affected by the mixture of multiple weather systems, so it is often inconsistent.

### Simulation results and response of different rain patterns in coastal areas

The experiment compares the runoff under different precipitation conditions, as shown in Table 3.

**Table 3:** Comparison of runoff under different rainfall conditions

Return period	Pre-peak rainfall peak flow (m3/s)			After peak rainfall peak flow (m3/s)		
	Traditional construction	LID measures	Peak reduction rate	Traditional construction	LID measures	Peak reduction rate
Once in 5 years	0.00074	0. 00061	21 .68%	0. 00087	0.0007	15.56%
Once in 20 years	0.00118	0. 00099	19.34%	0.00141	0.0012	13.79%
Once in 50 years	0.00163	0. 00143	13. 80%	0. 00183	0.0017	10.26%

Table 4 shows the peak discharge under different precipitation conditions.

**Table 4:** Comparison of peak discharge under different rainfall conditions

Return period	Pre-peak rainfall runoff (m3)			Rainfall runoff after peak (m3)		
	Traditional construction	LID measures	Runoff reduction rate	Traditional construction	LID measures	Runoff reduction rate
Once in 5 years	0. 0501	0. 0430	16.49%	0. 0591	0.0513	15.14%
Once in 20 years	0.0799	0.0718	1 l1.28%	0. 0925	0. 0838	10.38%
Once in 50 years	0.1102	0. 0997	10.50%	0.1138	1968-01-16	6.15%

Comparing the data in the table, it can be concluded that in different recovery periods, the current peak time has the same impact on the Runoff Effect of lid scale: peak flow and general flow. Under the same penetration rate, lid cascade structure level and turnover time, the control effect of lid cascade on runoff is usually better than that of previous precipitation. The most obvious difference was found in the 50 year recovery period, and the runoff reduction rate increased from 6.15% to 10.50%. After heavy rainfall, the maximum rainfall is usually much higher than the previous rainfall, and the peak rainfall is usually lower than the previous rainfall. This shows that lid has a positive effect on Regulating Runoff of pre peak rainfall.

**Fig. 5:**
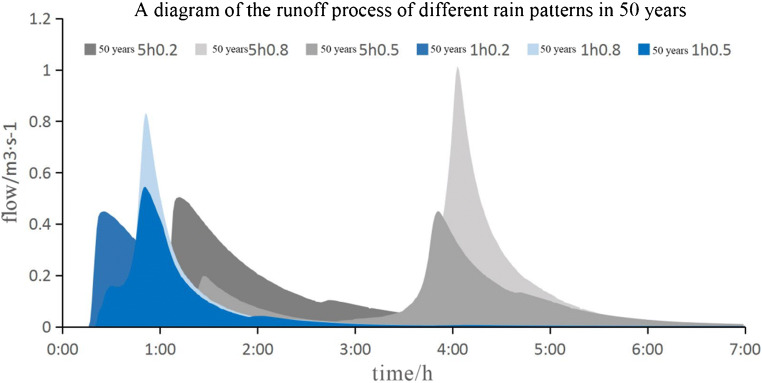
*P* = 5 and *P* = 50 runoff processes of different rain patterns

When *p* = 5 and *P* = 50, the different runoff maps of rainwater classification are shown in Fig. 5.

From the simulation results, it can be seen that for the same location of the precipitation peak, the longer the precipitation duration, the longer the peak increase time, the longer the return time, the larger the peak flow. In the same return period, the only maximum precipitation lasts for a long time, while the maximum precipitation lasts for a short time. The high bimodal rainfall is lower than the maximum rainfall. Comparing the different rainfall process maps of *P* = 5 and *P* = 50, the difference between the maximum precipitation after the peak precipitation and the total peak precipitation before the single peak precipitation and bimodal precipitation is almost twice, while the difference between the previous single peak precipitation and bimodal flow peak precipitation is very small. The peak height before *P* = 50 is relatively high. This shows that the lid step has the greatest influence on the double peak. If *P* = 5, lid measurement has the greatest influence on the peak shear of short-term pre peak precipitation. Generally, lid has no significant effect on the shear peak precipitation after the precipitation peak position.

**Fig. 6.**
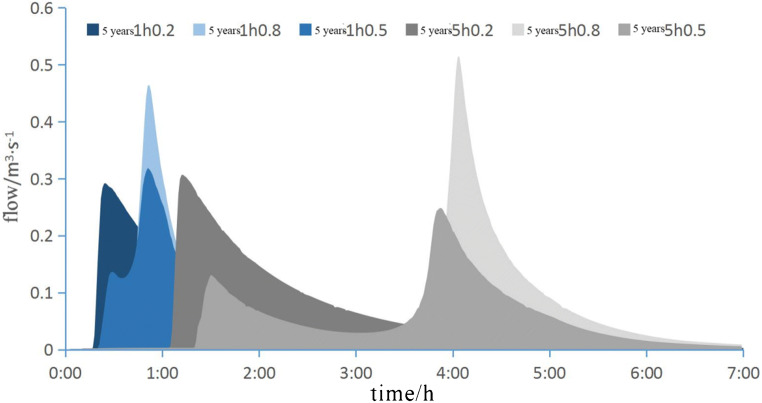
Compares the runoff processes of different rain patterns under the same return period (*P* = 5) and different saturation states (unsaturated and saturated)

For lid measurement, three different initial saturation states are established: unsaturated, semi saturated, and fully saturated. According to different initial saturation conditions, different return time and rainy season are simulated. Under the condition of precipitation, lid measurement results compare the operation of different precipitation models with the same return value (*P* = 5) and different saturation states (unsaturated and saturated). The operation process of the precipitation model is shown in Fig. 6.

During rainfall, the peak value of rainfall will come soon, and the rainwater from lid measures has no time to penetrate and absorb. Therefore, lid measures have adverse effects on runoff regulation of primary precipitation in semi saturated and fully saturated states.

### Influencing factors of different rain patterns in coastal areas

**Fig. 7.**
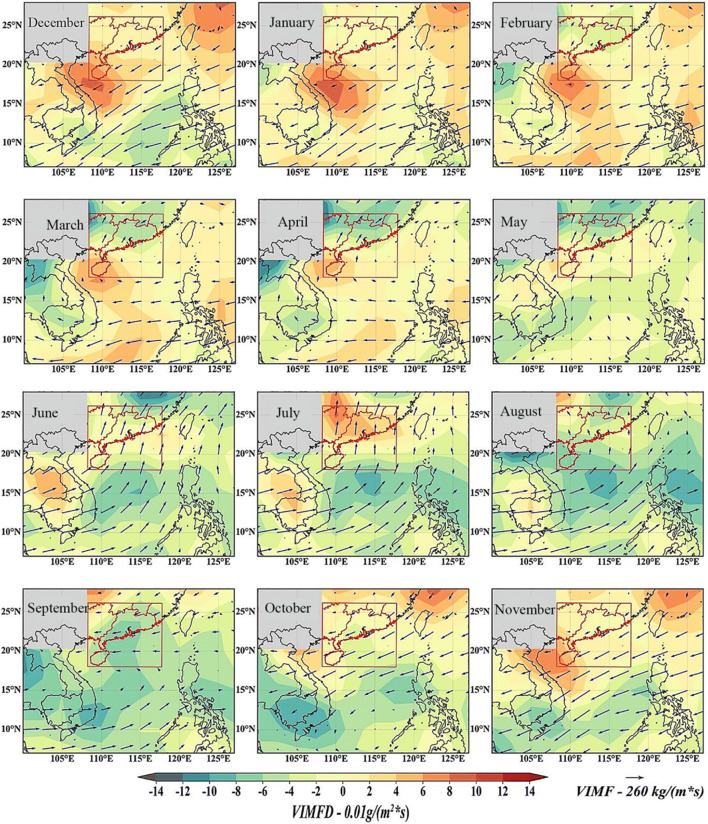
Spatial distribution of vertical integrated water vapor flux (VIMF) and vertical integrated water vapor flux divergence (vimfd) in South China

With the gradual closure of the summer water vapor channel, the summer water vapor in the southwest and south of the South China Sea gradually decreases, while the dry cold water vapor flows out from the northeast and gradually increases, resulting in the gradual weakening of autumn rainfall. Secondly, a typhoon circulation was found in the South China Sea in September. The westward flow from the Indian Ocean flows eastward through the bay of Bengal and produces a large amount of water vapor accumulation in the southern coastal area of H Province in China (Fig. 7). This has led to more rainfall in the area. In addition, heavy rainfall in H Province in September and October may be affected by tropical cyclones.

**Fig. 8.**
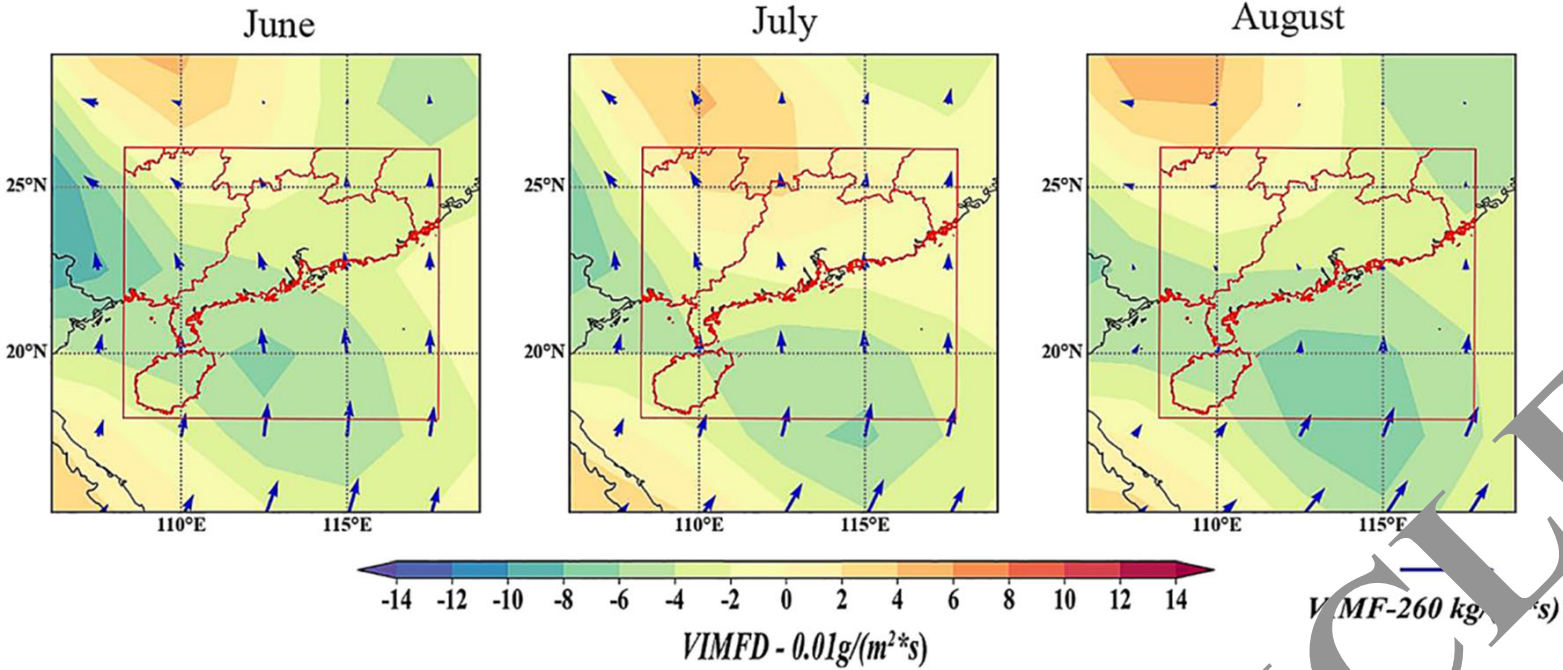
Spatial distribution of water vapor divergence and water vapor flux between 1000 HPA and 925 HPA in South China

In order to analyze the causes, the monthly water vapor budget of each pressure layer in southern China is calculated (see Fig. 8). It can be seen from Fig. 8 that the main area of water vapor budget is below 700 HPA, which further confirms the PT selected during the mixing layer. It can be seen from Fig. 8 that the strong change of water vapor in July shows the number of layers of 925hPa and 850hPa.

## Discussion

### Application of artificial intelligence in the development of cultural industry

The establishment of cultural industry can be divided into three categories: first, the establishment of cultural content and its carrier. The cultural content mainly includes words, images, audio, video, modeling, and comprehensive types. The carrier mainly consists of publications, raw materials, equipment, and facilities. The second is to provide cultural services, such as art performance, cultural creation, entertainment and cultural places, cultural fairs, cultural intermediaries, and vocational training; The third is the composition of the product, that is, the mixture of the first and second articles. In the production process of cultural industry, artificial intelligence can be competent in creating database and selecting theme, creating auxiliary or independent theme, modifying content, checking and correcting, changing operator, etc. The cultural creation of artificial intelligence has attracted the attention of the society (Ayoob and Gupta 2006).

AI link applications have the following differences: first, the Internet, Internet of things, and traditional media are migrated to the network structure of intelligent media, and intelligent management is used to reduce the lack of information, poor performance, and more connections between media, as well as idle and waste resources, sharing management, sharing services, etc. The transportation cost is reduced and the transportation speed is improved. Secondly, through intelligent social interaction, entertainment, communication can become lively and interesting, can take a variety of forms and can be accepted.

As the link to realize the value of cultural industry, consumption is the value of selling, trading, receiving, using experience, and feedback. The articles used are products with cultural connotation, cultural service products, and components. The AI application in this link will at least include the following contents: first, use AI technology to quickly and accurately analyze the needs of consumers, and then actively provide them with services such as content distribution, product recommendation, personal customization, and supply to meet their needs. Secondly, create a new, interesting, exciting, and practical smart grid application, such as photo editing, beauty, dubbing, karaoke, and so on, to attract many users to experiment and use (Azizullah et al. 2011). Master official account: Third, provide intelligent platform and tools for ordinary consumers to access the production and communication links of cultural industries, and help them become producers and broadcasters of audio and video, network writers, network anchors, micro-blog tattoo, and public numbers, as well as participants in cultural industries, crowd financing and construction activities (Baghani et al. 2017).

### Concept of global cultural communication

Chinese culture not only follows a single concept of communication. In order to achieve long-term development, we must adhere to the concept of communication diversity and integration, and create an environment conducive to the external communication of Chinese culture in the 21st century.

#### Guided by Marxism

Marxist ideology is not only the advanced theory guiding people to develop social life, but also the theoretical basis for people to develop culture. The main factor of Chinese culture communication is the opening of Chinese culture to the outside world. Marxist cultural thought is not only the basis of foreign cultural communication thought, but also includes doctrinal thought, in order to further strengthen the comprehensive development of culture, spread good Chinese culture and promote cooperation and cultural exchange. Marx said: “the globalization of production and communication will inevitably lead to the globalization of culture, which is the inevitable result.” Marxist cultural vision has always stressed the need to maintain tolerance and develop cultural virtues, and that the world is culturally diverse, only mutual learning and improvement can maintain the exchange of world civilization (Borysewicz-Lewicka and Opydo-Szymaczek 2016). Consistent with the progress of Marxist theory, the old state of self-isolation has been replaced by the status quo of mutual communication and trust among all nations “This is true of both material creation and spiritual creation. ” Therefore, to improve cultural exchange, we must follow the Marxist cultural view.

#### Conforming to the trend of Globalization

The dissemination of Chinese culture is an inevitable choice to adapt to the trend of globalization. The trend of globalization is promoting the interaction and exchange between countries and regions. Consistent with the analysis of globalization theory, globalization in modern civilization is an inevitable process of history. In a specific sense, economic globalization continues to promote the development of cultural globalization, and different countries have established their own values and world outlook in continuous exchanges (Chen et al. 2017). Cultural development must follow the law of global development, and strive to achieve “multicultural development system” in the process of cultural development. While maintaining different cultural differences while seeking common ground, “taking the essence and removing the dross” leads to a favorable environment conducive to the development of culture.

#### The spirit of the Chinese nation has rich connotation

In the context of modern culture, the spread of Chinese culture in the outside world has been inseparable from the spirit of the Chinese nation. With the smooth progress of reform and opening up and the continuous improvement of social process, the spirit of China’s times is of more and more importance, and more and more patriots are willing to add a part of their power. In the socialist modernization construction of the country, we should actively promote the socialist culture with the spirit of the times, and show the positive role of the spirit of the times in the process of cultural transmission.

The spirit of the Chinese nation can promote cultural exchanges (Choubisa 2018). For example, since ancient times, the spirit of the silk road has played a role in building a platform for cultural exchanges between East and West. The ancient Silk Road carried forward blue and white porcelain, calligraphy and painting, and craft culture. After the “one belt, one road” initiative promoted by the new era, China has actively and extensively worked with more than 60 countries and regions along the way, and has linked all other levels.

The national spirit of different periods also reflects the new development of Chinese culture in different periods, such as “four comprehensive” and “Beidou spirit in the new era” (Currell et al. 2011). The combination of different periods of cultural spirit and contemporary Chinese culture has made other countries have a more comprehensive understanding of the Chinese culture in the 21st century, which will help to promote the development of Chinese culture in the center of the world stage. Combining the spirit of China’s rich times with Chinese culture and spreading it all over the world through various channels will help to establish the image of China’s “great power”. China is committed to telling Chinese stories and promoting Chinese spirit through cultural exchanges, trade, and other channels to improve trade with other countries. The ancient spirit of the Chinese nation is the spiritual force necessary for the spread of culture, reflecting the vitality of Chinese culture in all countries around the world.

### Strategies of global cultural communication

At the beginning of the new century, China has carried out cultural cooperation and cultural exchanges with other countries in different ways, so that the people of many countries can feel the profound cultural heritage of China. As for the existing communication effect, there are still some areas to be improved in the process of external cultural communication. Looking for new methods of cultural communication can help to improve the current situation of cultural communication (Daiwile et al. 2018).

#### Give full play to the advantages of multiple communication subjects

Only when we have a comprehensive understanding of Chinese culture, can we pay attention to the characteristics of Chinese culture in foreign exchanges and improve the communication ability of excellent culture. We should give full play to the advantages of different cultural communication themes, constantly study the uniqueness of Chinese culture, and establish different cultural communication channels. China’s cultural departments and official media organizations give full play to various cultural and diplomatic functions, actively carry out cultural exchanges on a regular basis, emphasize the spirit of the Silk Road, and shape a good national image (Ding et al. 2011). Through official meetings and media and other forms to show the world the slow recovery of Chinese cultural power in the 21st century and actively promote the further integration of Chinese culture and the spirit of the times. Chinese netizens should express China’s positive voice on social media, make rational comments, avoid spreading rumors, and distinguish right from wrong. In addition, the channels of communication between overseas Chinese, overseas Chinese and patriots are very convenient, and Chinese culture can be better carried forward in various ways (Dutta et al. 2017). For example, traditional Chinese festivals and celebrations will continue to be held abroad, Chinese culture training institutions will be set up, public lectures on Chinese culture will be held, and Chinese food day will be established. It is necessary to spread China’s voice in different ways and establish a good image of China so that people of all countries can experience the unique beauty of Chinese culture in their lives.

In the process of spreading Chinese culture to foreign countries, we can also deepen cultural communication through academic exchanges, tourism projects, exhibitions of Chinese culture and history, and cooperation with Chinese and foreign education (Fallahzadeh et al. 2018). With the passage of time and the deepening of cultural communication, all social groups are encouraged to participate in the development of modern countries, spread the unique charm of Chinese culture to other countries and regions, and make the world better understand the state of synchronous development of Chinese culture.

#### Select the content that meets the needs of popular culture

The trend of globalization promotes multidimensional cooperation and participation in the process of mutual exchanges between countries, which is also the key condition for people to carry out cultural exchanges. There are differences in history, culture, economy, and national interests between countries, which also means that “users” have many differences, and their information needs, information choices, psychological acceptance, and use effects are also different (Ganyaglo et al. 2019). At present, the dissemination of Chinese culture pays more attention to the value of economy and trade, ignoring the national identity and the actual needs of other countries and regions (Huang et al. 2017). Cultural requirements and cultural values always complement each other. Only necessary culture can reflect its value. Therefore, in the process of spreading Chinese culture overseas, it is necessary to choose the traditional culture according to the local people’s values, and avoid the activities accepted by the local people just for the sake of dissemination. Numerous cooperation and sharing reflect the esthetic feeling of Chinese contemporary culture, and promote innovation and topicality according to the development needs of the times. Bring Chinese culture that can meet the needs of the people of all countries, make them understand the contemporary Chinese culture, spread Chinese culture more scientifically and reasonably, connect it with the world culture, and create a harmonious and unified cultural ecology (Guissouma et al. 2017).

#### Vigorously promote the construction of Chinese culture external communication talent team

There are obvious differences in language communication, cultural customs and economic conditions among countries, which hinder the establishment of “five links”. Language exchange is not only the most important factor in cultural exchange, but also an important bridge for international cultural exchange and an effective way to communicate with other countries (Khaliq et al. 2003). If we want to carry out in-depth cooperation with other countries, we should make foreign language talents the key direction of China’s personnel training. Foreign language computer skills can benefit from the process of foreign exchange. Foreign language talents with high level and excellent professional ability can have a great influence in international exchange and cooperation. In order to achieve the purpose of communication, foreign language communication must be based on different languages, but it is a process of cross-cultural communication. In addition to language skills, diplomats are also required to retain the knowledge of cultural communication (Mahvi et al. 2018). Domestic universities strictly abide by the “language and literature teaching quality standard” issued by the state in 2020, aiming at cultivating language talents. In terms of foreign languages, we should attach importance to the combination of knowledge and practice, speed up the cultivation of foreign language talents, and expand the spread of Chinese culture in the world. Language is not only the product of national development, but also carries the national culture (Lee et al. 2020).

With the development of globalization, many countries also have a strong interest in Chinese culture. Every country has its own works, language and customs, so Chinese teachers, scholars, and translators have become the mainstay of China’s external cultural communication. Nowadays, China mainly relies on the Confucius Institute as the main body of communication to strengthen the cooperation and exchange between countries and regions, so as to meet the needs of the world for Chinese culture. China should actively promote the construction of Chinese teachers, improve the Chinese learning system, so that people all over the world can better understand the richness and depth of Chinese culture in the process of learning Chinese.

## Conclusion

S city is located somewhere in the south of China. It is also the most populous city in the south of China. As an important city in China’s modernization and high-tech development, s city is vulnerable to bad weather, such as strong winds, short-term rainstorms, and storms. In recent years, due to the continuous increase of weather disasters, huge economic losses, and personnel losses have been caused in s city. Therefore, it is very important to study the spatial and temporal characteristics of rainfall distribution in South China, which plays a key role in supporting and maintaining the local flood control and drought relief policies. This study uses the latest meteorological observation data of 63 weather stations in South China from 1969 to 2020 to study the climate characteristics at the temporal and spatial level. Finally, this paper aims to study the issues related to global cultural communication. The research on world cultural communication will not only focus on new technology, but also on the connection of many disciplines and methods, and will focus on general research. In order to further improve the development of global cultural communication theory, we can find better communication channels from the perspective of discipline integration.
